# Impact of the triglyceride–glucose–neutrophil-to-lymphocyte ratio and the C-reactive protein–TyG index on cardio-renal disease in patients with type 2 diabetes

**DOI:** 10.3389/fendo.2025.1735949

**Published:** 2025-12-12

**Authors:** Ying Guo, Hongjian Jia, Yan Wang, Mengyan Li, Tong Chen, Zhendong Diao, Xicheng Li, Jietao Zhang

**Affiliations:** 1 The Affiliated Hospital of Jining Medical University, Jining, Shandong, China; 2 The Affiliated Hospital of Qingdao University, Qingdao, Shandong, China; 3 Shandong Second Medical University, Weifang, Shandong, China

**Keywords:** TyG-NLR, CTI, insulin resistance, systemic inflammation, ordinal severity, cardio-renal-metabolic disease

## Abstract

**Background:**

Insulin resistance and systemic inflammation jointly drive deterioration of cardiorenal-metabolic function; however, how composite indices reflect disease severity remains unclear. We compared the triglyceride–glucose–neutrophil-to-lymphocyte ratio (TyG-NLR) and a C-reactive protein–TyG–based index (CTI) to examine their associations with severity outcomes in hospitalized adults with type 2 diabetes.

**Methods:**

We conducted a retrospective study of hospitalized adults with type 2 diabetes at the Affiliated Hospital of Qingdao University, classifying cardiorenal disease into four severity levels. Using ordinal logistic regression, we evaluated the independent associations of the C-reactive protein–TyG–based index (CTI) and the triglyceride–glucose neutrophil-to-lymphocyte ratio (TyG-NLR) with severity, performed trend testing (p-trend), and explored potential nonlinearity with restricted cubic splines (RCS). For robustness, we additionally fitted a partial proportional odds model (VGAM framework) and a multinomial logistic model. Relative to a base covariate model, we assessed the incremental value of these indices in terms of overall model performance (AIC, BIC, Nagelkerke R², likelihood-ratio test), discrimination (AUC), calibration (calibration plot, slope, and calibration-in-the-large [CITL]), and clinical net benefit via decision-curve analysis (DCA).

**Results:**

A total of 2, 885 patients were included. In multivariable ordinal logistic regression analysis, both higher quartiles of CTI and TyG-NLR were significantly associated with increased disease severity (CTI_Q4 vs Q1: OR = 1.59, 95% CI 1.21–2.09; TyG-NLR_Q4 vs Q1: OR = 2.14, 95% CI 1.64–2.78; both P< 0.001). The Brant test indicated partial violation of the proportional odds assumption; sensitivity analysis using a VGAM-based partial proportional odds model yielded consistent results across thresholds. Trend tests revealed a significant linear increase in disease severity across quartiles for both indices (all P for trend < 0.001).Restricted cubic spline (RCS) analysis showed a nonlinear relationship between TyG-NLR and disease severity (LRT χ²= 34.438, P < 0.001), with the risk plateauing beyond a TyG-NLR value of approximately 16.64; in contrast, CTI exhibited an approximately linear association (LRT χ² = 1.486, P = 0.476). Regarding model performance, the TyG-NLR model achieved the best overall fit (AIC = 4367, BIC = 4488, Nagelkerke R² = 0.245, LR χ² = 50.8, P = 5.3 × 10^-^¹¹), while CTI yielded moderate improvement (LR χ² = 12.3, P = 0.006). In terms of discrimination, the TyG-NLR model attained the highest AUC of 0.680 (95% CI 0.668–0.693) and the lowest Brier score of 0.476. Calibration curves demonstrated good agreement at all thresholds (≥1, ≥2, ≥3), with the TyG-NLR model showing the closest alignment with the ideal line.Decision curve analysis (DCA) indicated that TyG-NLR provided the greatest net clinical benefit across a wide range of threshold probabilities (0.05–0.35), followed by CTI, while the incremental value of TyG alone was minimal. Both VGAM and multinomial logistic models yielded consistent directions of association, supporting the robustness of these findings.

**Conclusions:**

In adults with type 2 diabetes, both CTI and TyG-NLR were independently associated with cardiorenal disease severity.Notably, TyG-NLR demonstrated a steeper risk gradient and modest improvements in discrimination and calibration, and it yielded slightly higher net clinical benefit across clinically relevant decision thresholds. These findings suggest potential clinical utility for risk stratification, although the overall predictive gain was moderate and requires further validation.

## 1 Introduction

Cardiovascular disease (CVD) is one of the leading causes of mortality worldwide, with an incidence that continues to rise. This not only poses a serious threat to individuals’ health and quality of life but also presents a major challenge to public health systems (1–3). Its etiology is multifactorial, encompassing lifestyle behaviors, metabolic abnormalities, genetic predisposition, and underlying comorbidities (4–7). Cardiovascular disease and kidney disease are closely interrelated (8, 9), They exhibit a high degree of interdependence; cardio-renal interactions can precipitate a vicious spiral of organ dysfunction, marked by stepwise worsening of disease severity and a sharp increase in the risk of adverse outcomes. Accordingly, the American Heart Association (AHA) has integrated the cardiovascular and renal domains and introduced the concept of cardiovascular–kidney–metabolic (CKM) syndrome (10), The introduction of this concept has played an exceptional role in preventing disease onset, mitigating or slowing disease progression, and improving clinical outcomes. It underscores the necessity of investigating cardiovascular disease in conjunction with renal and metabolic disorders.

The triglyceride–glucose index (TyG) is widely regarded as a simple surrogate marker of insulin resistance (11); Research on the TyG index in cardiovascular and cerebrovascular diseases has expanded rapidly in recent years, confirming its important role in disease onset, progression, and prognosis (12–14). Compared with the euglycemic–hyperinsulinemic clamp or the homeostatic model assessment of insulin resistance (HOMA-IR), the TyG index can be calculated in virtually all clinical settings at minimal additional cost, and it has been closely associated with the incidence of cardiovascular and renal events. Therefore, the TyG index and TyG-based composite indicators offer a practical approach for detecting subclinical insulin resistance not only in patients with established type 2 diabetes, but also in apparently non-diabetic individuals who may already carry an excessive cardio-renal risk burden.The neutrophil-to-lymphocyte ratio (NLR) reflects systemic inflammation and immune status (15). The TyG-NLR, formed by the combination of the two, holds significant importance for evaluating metabolic disorders and inflammatory responses. Several studies have shown that metabolic imbalance and inflammatory responses act in concert to exacerbate atherosclerosis, myocardial remodeling, and renal injury (16). Research on composite indices incorporating the TyG index has deepened in recent years. Combinations of TyG with measures such as waist circumference (WC), body mass index (BMI), and waist-to-height ratio (WHtR) are widely used to predict and evaluate the onset and prognosis of cardiovascular disease, and these combined indices provide greater discriminative value than the TyG index alone. The C-reactive protein–triglyceride–glucose index (CTI) integrates information on lipid status, glucose metabolism, and inflammation, and shows potential predictive value for cardiovascular events and deterioration of renal function (17, 18).

Most existing studies have focused on binary outcomes (“presence or absence” of disease), while investigations addressing the ordered progression of disease severity remain limited. Moreover, comparative studies of the two composite indices—TyG-NLR and CTI—within the same population, using *disease severity* as a shared clinical endpoint, are scarce. This gap has hindered their broader application in practical risk stratification and model enhancement evaluation.

Based on this evidence gap, the present study aims to systematically assess the associations of TyG-NLR and CTI with the severity of cardiorenal disease in a real-world clinical population. By employing “Severity of Disease” as an outcome more closely aligned with clinical decision-making, this study seeks to validate and compare the value of these two easily accessible composite indices in cardiorenal risk assessment, thereby laying a methodological and evidential foundation for future multicenter and population-based studies.

## 2 Methods

### 2.1 Study design and population

Data for this study were obtained from the electronic medical records (EMR) system of the Affiliated Hospital of Qingdao University. We identified hospitalized adults with type 2 diabetes mellitus (T2DM) who met the following inclusion criteria: (1) age ≥18 years; (2) a confirmed diagnosis that included diabetes, cardiovascular disease, and renal disease; (3) complete clinical and laboratory data; (4) non-pregnant status; and (5) absence of malignancy or other cachectic conditions.

From the EMR, we extracted demographic information (age, sex, body mass index [BMI]); medical history (hypertension, smoking, alcohol consumption); laboratory parameters (fasting plasma glucose, lipid profile, uric acid, albumin, inflammatory markers, hemoglobin); and medication use (statins, insulin, sodium–glucose cotransporter-2 inhibitors [SGLT2i]).

### 2.2 Variables and outcome definitions

The study outcome was “Severity of Disease” defined as an ordinal variable representing the extent of cardiorenal involvement: 0 = no cardiovascular disease (CVD) or chronic kidney disease (CKD); 1 = CKD only; 2 = CVD only; 3 = coexistence of CVD and CKD.

Covariates included sex, age, smoking and alcohol status, body mass index (BMI), use of statins and sodium–glucose cotransporter-2 inhibitors (SGLT2i), hypertension, insulin use, total cholesterol (TC), low-density lipoprotein cholesterol (LDL-C), high-density lipoprotein cholesterol (HDL-C), albumin, uric acid (UA), and hemoglobin.

Inflammation–metabolic composite indices are calculated as follows:


TYG index=ln[(TG(mg/dL)×FPG(mg/dL))/2]

NLR=neutrophil count/lymphocyte count

TYG−NLR=TyG×NLR

For comparability, the continuous exposures (CTI and TyG-NLR) were analyzed both in their original continuous form and by quartiles (Q1–Q4). Where appropriate, routine biochemical variables with marked skewness were log-transformed or standardized as z-scores.

### 2.3 Statistical analysis

#### 2.3.1 Descriptive analysis

Normality was assessed using the Shapiro–Wilk test. Continuous variables were expressed as mean ± standard deviation (SD) for normally distributed data or median (interquartile range) for skewed data. Between-group differences were evaluated using one-way analysis of variance (ANOVA) or the Kruskal–Wallis test, respectively. Categorical variables were presented as frequencies and percentages and compared using the chi-square or Fisher’s exact test. Multicollinearity was tested using the variance inflation factor (VIF < 5 as acceptable).

#### 2.3.2 Main regression models

The association between each composite index and disease severity was first examined using the proportional-odds (PO) ordinal logistic regression model, with full adjustment for covariates. The Brant test was applied to verify the PO assumption. When partial violation occurred, a partial proportional-odds model (fitted with the VGAM package) was used, allowing threshold-specific coefficients. Sensitivity analyses included multinomial logistic regression to confirm consistency in effect direction and magnitude.

#### 2.3.3 Trend and dose–response analysis

For quartile-based exposures, linear trends across categories were tested using both: the median value of each quartile as a continuous variable (main analysis), and an ordinal score (1–4) assigned to quartiles (sensitivity analysis).

Significance was evaluated using Wald and likelihood ratio tests (p-trend).

For continuous exposures, restricted cubic splines (RCS) with four knots at empirical percentiles were used to explore dose–response and potential nonlinearity. Nonlinearity was assessed using likelihood ratio tests comparing spline and linear models. Odds ratios (ORs) and 95% confidence intervals (CIs) were computed for the 10th, 25th, 50th, 75th, and 90th percentiles.

#### 2.3.4 Model performance evaluation

To assess incremental predictive value, model fit (AIC, BIC, Nagelkerke R², likelihood ratio χ²), discrimination (area under the curve [AUC]), and calibration (calibration plots, slope, and calibration-in-the-large [CITL]) were compared between base and augmented models.

Decision curve analysis (DCA) was performed to quantify the net clinical benefit across threshold probabilities ranging from 0.05 to 0.35.

#### 2.3.5 Missing data and significance thresholds

A complete-case approach was applied without imputation. All statistical tests were two-sided, and *P* < 0.05 was considered statistically significant.

#### 2.3.6 Software

All analyses were performed using R version 4.5.0. Packages used included rms, ordinal, MASS, VGAM, brant, pROC, boot, and dcurves (or rmda).

### 2.4 Ethical considerations

This research protocol has been approved by the Affiliated Hospital of Qingdao University. As this study is retrospective in nature, informed consent is not required. The research complies with the Declaration of Helsinki.

## 3 Results

### 3.1 Baseline characteristics

This study included 2, 885 participants, of whom 1, 798 (62.32%) had a Severity of Disease score of 0, 382 (13.24%) had a score of 1, 529 (18.34%) had a score of 2, and 176 (6.10%) had a score of 3. Statistically significant differences were observed between the four groups for Age, BMI, Total Cholesterol (TC), Albumin, Low-Density Lipoprotein Cholesterol (LDL-C), Uric Acid (UA), Haemoglobin, TyG Index, Neutrophil-to-Lymphocyte Ratio (NLR), C-reactive protein-TyG Index (CTI), Statin Use, Hypertension, and Insulin (P < 0.05). Conversely, no statistically significant differences were found for High-Density Lipoprotein Cholesterol (HDL-C), Sex, Smoking Status, Alcohol Consumption, or Sodium–glucose cotransporter-2 inhibitor (SGLT2i) Use (P > 0.05). See **Table 1** for details.

**Table 1:** Baseline characteristics of hospitalised adult patients with type 2 diabetes in the classification of cardiovascular and renal disease severity.

Variables	Total (n = 2885)	0 (n = 1798)	1 (n = 382)	2 (n = 529)	3 (n = 176)	Statistic	P-value
age, Mean ± SD	64.39 ± 12.17	63.08 ± 11.72	63.91 ± 14.32	66.37 ± 10.58	72.81 ± 12.06	F=41.52	<.001
BMI, Mean ± SD	26.74 ± 43.83	26.07 ± 13.87	25.99 ± 10.30	25.57 ± 7.89	39.37 ± 174.58	F=4.38	0.004
TC, Mean ± SD	4.18 ± 1.51	4.12 ± 1.34	4.67 ± 2.11	4.05 ± 1.27	4.23 ± 2.01	F=15.86	<.001
Albumin, Mean ± SD	45.66 ± 10.34	46.96 ± 9.80	41.74 ± 12.05	45.24 ± 9.50	42.14 ± 11.02	F=35.87	<.001
LDL_C, Mean ± SD	2.50 ± 1.03	2.45 ± 0.89	2.87 ± 1.52	2.39 ± 0.86	2.50 ± 1.39	F=19.88	<.001
UA, Mean ± SD	334.02 ± 109.52	314.17 ± 94.28	395.59 ± 121.99	324.93 ± 107.02	433.38 ± 131.53	F=120.64	<.001
HDL_C, Mean ± SD	1.21 ± 0.34	1.22 ± 0.34	1.20 ± 0.38	1.19 ± 0.32	1.17 ± 0.33	F=1.71	0.162
Hemoglobin, Mean ± SD	124.33 ± 30.16	127.97 ± 26.65	108.51 ± 39.88	129.18 ± 27.94	106.80 ± 28.53	F=73.30	<.001
TyG NLR Q, n(%)						χ²=161.67	<.001
Q1	722 (25.03)	528 (29.37)	61 (15.97)	119 (22.50)	14 (7.95)		
Q2	721 (24.99)	492 (27.36)	83 (21.73)	123 (23.25)	23 (13.07)		
Q3	721 (24.99)	418 (23.25)	85 (22.25)	160 (30.25)	58 (32.95)		
Q4	721 (24.99)	360 (20.02)	153 (40.05)	127 (24.01)	81 (46.02)		
CTI Q, n(%)						χ²=141.24	<.001
Q1	722 (25.03)	532 (29.59)	53 (13.87)	115 (21.74)	22 (12.50)		
Q2	721 (24.99)	463 (25.75)	67 (17.54)	157 (29.68)	34 (19.32)		
Q3	721 (24.99)	425 (23.64)	105 (27.49)	145 (27.41)	46 (26.14)		
Q4	721 (24.99)	378 (21.02)	157 (41.10)	112 (21.17)	74 (42.05)		
Sex, n(%)						χ²=2.84	0.417
1	1293 (44.82)	818 (45.49)	156 (40.84)	239 (45.18)	80 (45.45)		
2	1592 (55.18)	980 (54.51)	226 (59.16)	290 (54.82)	96 (54.55)		
Smoke, n(%)						χ²=1.31	0.728
0	2118 (73.72)	1328 (73.94)	282 (75.20)	380 (71.97)	128 (73.56)		
1	755 (26.28)	468 (26.06)	93 (24.80)	148 (28.03)	46 (26.44)		
Drink, n(%)						χ²=7.48	0.058
0	2212 (77.02)	1360 (75.85)	304 (80.64)	404 (76.52)	144 (82.76)		
1	660 (22.98)	433 (24.15)	73 (19.36)	124 (23.48)	30 (17.24)		
Statin, n(%)						χ²=107.94	<.001
0	1514 (52.48)	1027 (57.12)	235 (61.52)	194 (36.67)	58 (32.95)		
1	1371 (47.52)	771 (42.88)	147 (38.48)	335 (63.33)	118 (67.05)		
SGLT2i, n(%)						χ²=1.76	0.623
0	2810 (97.40)	1751 (97.39)	369 (96.60)	517 (97.73)	173 (98.30)		
1	75 (2.60)	47 (2.61)	13 (3.40)	12 (2.27)	3 (1.70)		
Hypertension, n(%)						χ²=408.89	<.001
0	2532 (87.76)	1697 (94.38)	362 (94.76)	378 (71.46)	95 (53.98)		
1	353 (12.24)	101 (5.62)	20 (5.24)	151 (28.54)	81 (46.02)		
Insulin, n(%)						χ²=40.60	<.001
0	2481 (86.00)	1568 (87.21)	299 (78.27)	478 (90.36)	136 (77.27)		
1	404 (14.00)	230 (12.79)	83 (21.73)	51 (9.64)	40 (22.73)		

F, ANOVA; χ², Chi-square test; SD, standard deviation.

### 3.2 Ordinal logistic regression and proportional odds assumption

When the CTI was categorized into quartiles (Q1 as the reference group) and adjusted for covariates including sex, age, smoking, alcohol consumption, BMI, statin use, SGLT2i use, hypertension, insulin use, total cholesterol, albumin, LDL-C, HDL-C, uric acid, and hemoglobin, CTI was found to be significantly positively associated with the Severity of Disease (**Table 2**).As CTI increased, the risk of higher disease severity rose progressively:Q2 vs Q1: OR = 1.28 (95% CI: 0.99–1.67, P = 0.061);Q3 vs Q1: OR = 1.46 (95% CI: 1.12–1.91, P = 0.005);Q4 vs Q1: OR = 1.59 (95% CI: 1.21–2.09, P < 0.001).

**Table 2:** Multivariable ordinal logistic regression analysis for the association between CTI quartiles and cardiorenal disease severity.

Variables	Level	OR (95%CI)	P-value
CTI	Q2	1.28 (0.99, 1.67)	0.061
CTI	Q3	1.46 (1.12, 1.91)	0.005
CTI	Q4	1.59 (1.21, 2.09)	< 0.001
sex		1.12 (0.91, 1.39)	0.286
age		1.03 (1.02, 1.04)	< 0.001
Smoke		1.01 (0.76, 1.33)	0.965
Drink		0.82 (0.61, 1.09)	0.166
BMI		1.00 (1.00, 1.00)	0.063
Statin		1.58 (1.31, 1.90)	< 0.001
SGLT2i		1.06 (0.61, 1.85)	0.827
Hypertension		5.92 (4.54, 7.72)	< 0.001
Insulin		0.79 (0.61, 1.02)	0.071
TC		0.96 (0.86, 1.07)	0.457
Albumin		0.97 (0.96, 0.98)	< 0.001
LDL_C		1.05 (0.91, 1.22)	0.510
UA		1.00 (1.00, 1.01)	< 0.001
HDL_C		1.17 (0.86, 1.57)	0.315
Hemoglobin		0.99 (0.99, 1.00)	< 0.001

Using the same approach, TyG-NLR showed a significant positive association with disease severity (**Table 3**). Compared with Q1, the risk increased progressively across quartiles:Q2 vs Q1: OR = 1.31 (95% CI: 1.02–1.68, P = 0.034); Q3 vs Q1: OR = 1.64 (95% CI: 1.26–2.13, P < 0.001); Q4 vs Q1: OR = 2.10 (95% CI: 1.56–2.82, P < 0.001).

**Table 3:** Multivariable ordinal logistic regression analysis for the association between TyG-NLR quartiles and cardiorenal disease severity.

Variables	Level	OR (95%CI)	P-value
TyG-NLR	Q2	1.10 (0.84, 1.42)	0.491
TyG-NLR	Q3	1.88 (1.46, 2.42)	< 0.001
TyG-NLR	Q4	2.14 (1.64, 2.78)	< 0.001
sex		1.01 (0.81, 1.25)	0.964
age		1.02 (1.02, 1.03)	< 0.001
Smoke		1.00 (0.76, 1.32)	0.990
Drink		0.83 (0.62, 1.11)	0.201
BMI		1.00 (1.00, 1.00)	0.041
Statin		1.63 (1.36, 1.96)	< 0.001
SGLT2i		1.15 (0.66, 2.01)	0.622
Hypertension		5.72 (4.38, 7.46)	< 0.001
Insulin		0.75 (0.58, 0.98)	0.032
TC		0.97 (0.87, 1.08)	0.555
Albumin		0.97 (0.96, 0.98)	< 0.001
LDL_C		1.09 (0.94, 1.26)	0.243
UA		1.00 (1.00, 1.01)	< 0.001
HDL_C		1.08 (0.81, 1.44)	0.605
Hemoglobin		0.99 (0.99, 1.00)	< 0.001

The proportional odds (parallel lines) assumption for both models was evaluated using the Brant test. For the CTI quartile model, the overall Brant test indicated that the proportional odds assumption was not fully satisfied. Variable-specific testing further revealed that the CTI quartile levels significantly violated this assumption.

Similarly, the TyG-NLR quartile model showed partial deviations from the proportional odds assumption. Variable-specific results indicated that higher TyG-NLR quartiles exhibited significant departures from proportionality. Details are shown in **Table 4**.

**Table 4:** Brant test results for proportional odds (PO) assumption in ordinal logistic regression models of CTI and TyG-NLR.

Variables	χ2	df	P-value
Omnibus	670.1024343	36	< 0.001
CTI_Q2	3.025500971	2	0.220
CTI_Q3	0.336024179	2	0.845
CTI_Q4	4.660314225	2	0.097
Omnibus	645.194242	36	< 0.001
TyG_NLR_Q2	2.963367059	2	0.227
TyG_NLR_Q3	7.700501831	2	0.021
TyG_NLR_Q4	14.90324521	2	< 0.001

Omnibus and variable-specific tests were performed to assess the proportional-odds assumption. P < 0.05 indicates violation of the assumption.

### 3.3 Partial proportional odds (VGAM) analysis

Because the overall Brant test did not fully support the proportional odds assumption, we performed a sensitivity analysis using a partial proportional odds model (VGAM). The results showed that the threshold-specific odds ratios for CTI and TyG-NLR were directionally consistent and exhibited stable trends across cutpoints, indicating that the primary model findings are robust. (**Table 5**).

**Table 5:** Partial proportional odds model (VGAM) results for CTI and TyG-NLR with threshold-specific coefficients.

Assumption (PO)	Variables	Exposure	
		CTI	TyG-NLR
		OR (95%CI)	
Parallel (PO)	age	0.98 (0.97, 0.99)	0.98 (0.97, 0.99)
Parallel (PO)	Albumin	1.02 (1.02, 1.03)	1.02 (1.01, 1.03)
Parallel (PO)	BMI	1.00 (1.00, 1.00)	1.00 (1.00, 1.00)
Parallel (PO)	Drink	1.17 (0.88, 1.56)	1.16 (0.87, 1.53)
Parallel (PO)	HDL_C	0.89 (0.66, 1.21)	0.96 (0.72, 1.28)
Parallel (PO)	Hemoglobin	1.01 (1.00, 1.01)	1.00 (1.00, 1.01)
Parallel (PO)	Hypertension	0.20 (0.15, 0.26)	0.21 (0.16, 0.27)
Parallel (PO)	Insulin	1.21 (0.93, 1.59)	1.25 (0.96, 1.63)
Parallel (PO)	LDL_C	0.94 (0.81, 1.10)	0.92 (0.79, 1.06)
Parallel (PO)	sex	0.91 (0.73, 1.12)	1.00 (0.81, 1.24)
Parallel (PO)	SGLT2i	0.90 (0.52, 1.55)	0.84 (0.48, 1.45)
Parallel (PO)	Smoke	1.00 (0.76, 1.32)	1.01 (0.77, 1.33)
Parallel (PO)	Statin	0.68 (0.57, 0.82)	0.66 (0.55, 0.80)
Parallel (PO)	TC	1.05 (0.94, 1.17)	1.03 (0.93, 1.15)
Parallel (PO)	UA	1.00 (0.99, 1.00)	1.00 (0.99, 1.00)

The partial proportional odds model (VGAM framework) was fitted to account for non-parallel slopes. Results show stable direction and magnitude across thresholds, supporting robustness.

### 3.4 Trend analysis

Trend tests (*p*-trend) demonstrated a significant linear relationship between CTI and disease severity (median method: Wald *P* = 8.2×10^-4^; LRT *P* = 8.2×10^-4^; score method: Wald *P* = 7.3×10^-4^; LRT *P* = 7.3×10^-4^). A highly significant increasing trend was also observed between TyG-NLR and disease severity (median method: Wald *P* = 5.3×10^-11^; LRT *P* = 6.6×10^-11^; score method: Wald *P* = 1.5×10^-^¹¹; LRT *P* = 1.2×10^-^¹¹).

### 3.5 Restricted cubic splines

In the RCS models, TyG-NLR showed a significant nonlinear association with the outcome (LRT for nonlinearity: χ² = 34.438, df = 2, P = 3.33×10^-8^). The odds ratio (OR) for TyG-NLR was 0.70 (95% CI: 0.56–0.87) at the 10th percentile, 1.36 (1.12–1.65) at the 75th percentile, and 1.76 (1.37–2.27) at the 90th percentile (**Table 6**).

**Table 6:** Restricted cubic spline (RCS) analysis of dose–response relationships between TyG-NLR, CTI, and cardiorenal disease severity.

Exposure	Percentile	Value	OR (95% CI)
TyG_NLR	10%	9.2977	0.70 (0.56, 0.87)
TyG_NLR	25%	12.1815	0.81 (0.67, 0.98)
TyG_NLR	50% (Ref)	16.6387	1.00 (0.82, 1.21)
TyG_NLR	75%	25.1863	1.36 (1.12, 1.65)
TyG_NLR	90%	40.9329	1.76 (1.37, 2.27)
CTI	10%	7.8287	0.73 (0.58, 0.92)
CTI	25%	8.2902	0.87 (0.70, 1.10)
CTI	50% (Ref)	8.9150	1.00 (0.83, 1.21)
CTI	75%	9.5168	1.08 (0.87, 1.33)
CTI	90%	10.1238	1.17 (0.95, 1.44)

Odds ratios (ORs) and 95% CIs were computed at the 10th, 25th, 50th, 75th, and 90th percentiles. LRT: likelihood ratio test for nonlinearity.

By contrast, the exposure–response relationship for CTI was approximately linear, with no evidence of significant nonlinearity (LRT: χ² = 1.486, df = 2, P = 0.476). Its risk increased gradually with higher CTI (see figure “RCS for CTI”): ORs were 0.73 (0.58–0.92) at the 10th percentile, 1.08 (0.87–1.33) at the 75th percentile, and 1.17 (0.95–1.44) at the 90th percentile (**Table 6**).

**Figure 1:**
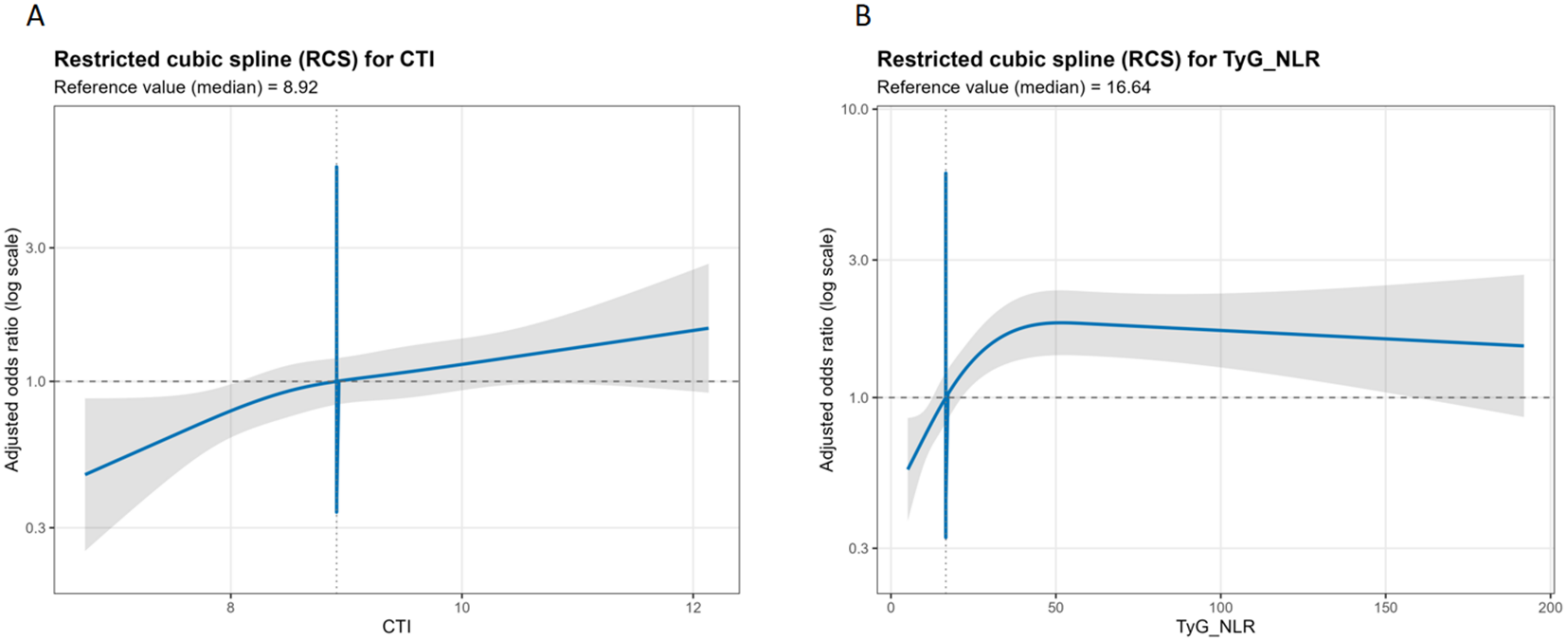
Restricted cubic spline (RCS) curves for the associations of **(A)** TyG-NLR and **(B)** CTI with cardiorenal disease severity. Solid lines represent adjusted odds ratios; shaded areas indicate 95% confidence intervals. The horizontal dashed line marks OR = 1, and the vertical dashed line denotes the median exposure. TyG-NLR displays a nonlinear association plateauing beyond 16.6, while CTI shows a linear relationship.

The exposure–response relationships between TyG-NLR and CTI and cardiorenal disease severity are illustrated in **Figure 1**.

### 3.6 Model performance comparison

**Figure 2:**
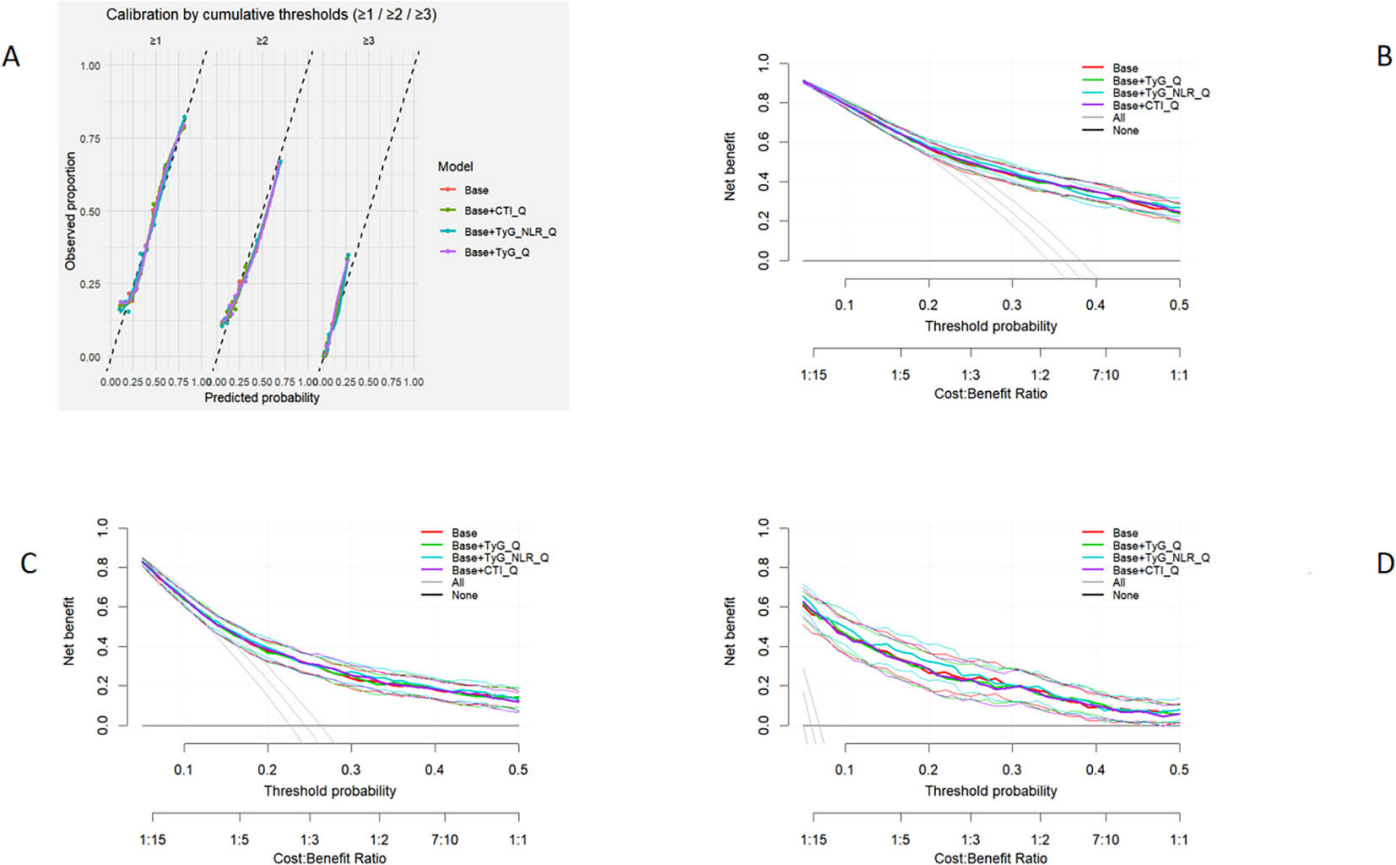
Calibration and decision-curve analyses of models incorporating TyG, TyG-NLR, and CTI for predicting cardiorenal disease severity in patients with type 2 diabetes. **(A)** Calibration plots at cumulative thresholds (≥1, ≥2, ≥3) showing agreement between predicted and observed probabilities across models. **(B–D)** Decision-curve analyses demonstrating net clinical benefit of each model over a range of threshold probabilities. The TyG-NLR-augmented model achieved the best calibration and yielded the greatest net benefit across threshold probabilities of 0.05–0.35, followed by CTI, while TyG alone provided minimal incremental value. Base = covariate-only model; CTI =C-reactive-protein–triglyceride–glucose;TyG-NLR=triglyceride–glucose–neutrophil-to-lymphocyte ratio.

As shown in **Table 7**, compared with the base model, inclusion of TyG-NLR (Base+TyG-NLR) substantially improved overall model performance (AIC = 4367, BIC = 4488, Nagelkerke R² = 0.245, LR χ² = 50.8, P = 5.3×10^-^¹¹). The Base + CTI model demonstrated moderate improvement (LR χ² = 12.3, P = 0.006), whereas the TyG model did not yield a significant gain (P = 0.23). Among all models, the TyG-NLR model achieved the highest AUC (0.680, 95% CI: 0.668–0.693) and the lowest Brier score (0.476). Calibration curves indicated good agreement between predicted and observed probabilities across all thresholds (≥1, ≥2, ≥3), with the TyG-NLR model showing the closest alignment with the ideal calibration line. Minor deviations in slope and CITL suggested slight overprediction at higher thresholds (**Figure 2A**). Decision curve analysis (DCA) further demonstrated that the TyG-NLR model consistently provided the greatest net clinical benefit over a wide range of threshold probabilities (0.05–0.35), followed by the CTI model, while the TyG model offered the least additional value. (**Figures 2B-D**).

**Table 7:** Model performance comparison for base, TyG, TyG-NLR, and CTI models.

Model	AIC	BIC	LogLik	Nagelkerke R²	AUC (95% CI)	Brier	Brier scaled	LR test vs Base (χ², df, P)	Calibration slope (≥1/≥2/≥3)	CITL (≥1/≥2/≥3)
Base	4412.1	4515.7	−2188.0	0.225	0.675 (0.661–0.690)	0.481	0.109	—	0.96/0.82/1.40	0.006/−0.013/−0.028
Base + TyG_Q	4413.2	4535.0	−2186.3	0.227	0.676 (0.661–0.692)	0.481	0.110	4.31, 3, 0.230	0.97/0.82/1.40	0.006/−0.012/−0.028
Base + TyG-NLR_Q	4367.3	4488.4	−2162.4	0.245	0.680 (0.668–0.693)	0.476	0.118	50.8, 3, 5.3×10^-^¹¹	0.97/0.81/1.43	0.007/−0.013/−0.031
Base + CTI_Q	4405.1	4527.3	−2182.3	0.230	0.677 (0.663–0.692)	0.480	0.111	12.3, 3, 0.006	0.97/0.82/1.41	0.006/−0.012/−0.028

Model performance was evaluated by Akaike information criterion (AIC), Bayesian information criterion (BIC), Nagelkerke R², area under the ROC curve (AUC), Brier score, and calibration metrics. LR χ² tests assessed incremental improvement versus the base model.

## 4 Discussion

This study stratified type 2 diabetes mellitus (T2DM) patients according to the CKM staging system to further categorize their cardiorenal status and investigate the effects of the TyG-NLR and CTI indices on cardiorenal function. Using real-world inpatient data in a retrospective design, we found that composite indicators reflecting systemic inflammation and insulin resistance were independently associated with the severity of cardiorenal impairment in this population. Notably, TyG-NLR exhibited a relatively steeper risk gradient and modest improvements in discrimination and calibration, and it yielded slightly higher net clinical benefit across a range of decision thresholds. Given that the absolute AUC remained in the moderate range and the increase in Nagelkerke R² was small, the added value of TyG-NLR should be interpreted as modest but potentially useful for early risk assessment.

Inflammatory markers play a crucial role in cardiovascular disease, a concept that has been well established in numerous previous studies, For example, Kaptoge S and colleagues found that C-reactive protein (CRP) concentrations are consistently associated with the development and prognosis of cardiovascular disease (19). Xia and colleagues likewise reported that the systemic immune-inflammation index (SII) and the systemic inflammation response index (SIRI) are closely associated with cardiovascular mortality and all-cause mortality (20); In addition, the study by Haffner SM also substantiated the impact of inflammatory status on cardiovascular outcomes (21). In a prognostic study of heart failure, the NLR was identified as a practical tool for risk stratification and screening of high-risk patients in routine clinical practice (22). The mechanisms by which inflammation leads to cardiac injury are likely multifactorial. Inflammatory cytokines can induce cardiomyocyte apoptosis and even contribute to ventricular remodeling (23);In addition, inflammatory responses can disrupt lipid metabolism for example, by impairing reverse cholesterol transport thereby diminishing anti-atherosclerotic capacity and further accelerating the onset and progression of cardiovascular disease (24).

Insulin resistance (IR) also exerts an indispensable impact on cardiorenal disease. The onset of IR is often accompanied by dysregulation of metabolic factors, such as blood lipids and glucose that are established risk factors for cardiorenal disorders (25);Concurrently, IR impairs vascular vasodilation and exacerbates arteriosclerosis (26);IR also activates inflammatory responses (27), which, together with its vascular effects, promotes plaque formation. Insulin resistance (IR) is not only a metabolic abnormality but also a direct driver of cardiovascular and renal injury. First, IR induces endothelial dysfunction by reducing nitric oxide bioavailability, increasing oxidative stress, and enhancing endothelin-1 activity, thereby impairing vasodilation (28, 29). These alterations accelerate arterial stiffness and the formation of atherosclerotic plaques. Second, IR disrupts reactive oxygen species (ROS) homeostasis in the myocardium, leading to cumulative superoxide-mediated injury or cellular dysfunction. This imbalance contributes to mitochondrial stress, cardiomyocyte hypertrophy, and interstitial fibrosis, ultimately resulting in adverse cardiac remodeling and a heart failure phenotype (30). In the kidney, insulin and hyperinsulinemia increase tubular sodium reabsorption and glomerular hyperfiltration, creating intraglomerular hypertension and mesangial expansion, which in turn promote proteinuria and progressive loss of renal function (31).Moreover, IR activates the sympathetic nervous system and the renin–angiotensin–aldosterone system, causing elevated blood pressure, increased volume load, and further hemodynamic stress on both the heart and kidneys. IR is also accompanied by low-grade chronic inflammation; adipose-derived cytokines such as TNF-α and IL-6 impair insulin signaling while fostering a pro-inflammatory and pro-thrombotic microenvironment. In recent years, research on the surrogate index TyG has deepened, and its combinations with other risk factors have markedly improved discrimination for diagnosis, incidence, and prognosis—e.g., TyG-BMI (32), TyG- waist circumference (33), TyG-ABSI (34). Accordingly, TyG-NLR integrates metabolic and immuno-inflammatory dimensions into a single construct. We found that higher TyG-NLR quartiles were closely associated with increased disease severity (Q4 vs Q1 OR = 2.14), supporting the notion that synergistic metabolic–inflammatory stress may accelerate injury to cardiac and renal tissues. This composite mechanism may partly explain the observed nonlinear exposure–response curve: risk rises steeply up to approximately TyG-NLR ≈ 16.6 and then plateaus, suggesting saturation of pathophysiological burden. By contrast, CTI exhibited an approximately linear dose–response relationship, indicating a more stable—and less dynamic—reflection of systemic inflammation. Although CTI was also significantly associated with disease severity (Q4 vs Q1 OR = 1.59), its overall discriminative ability was inferior to that of TyG-NLR.

Compared with previous studies, our findings extend the existing evidence linking TyG and NLR separately to cardiovascular and renal outcomes (15, 35–37). By evaluating the composite indices TyG-NLR and CTI against an ordered severity endpoint and comparing them within a unified analytic framework, we show that TyG-NLR may outperform CTI in quantifying the integrated metabolic inflammatory burden associated with cardiorenal outcomes.

Our decision curve analysis demonstrated that TyG-NLR provided meaningful net clinical benefits within a threshold probability range of 0.05–0.35—a range commonly encountered in real-world screening and early intervention decision-making scenarios. Therefore, incorporating TyG-NLR into electronic medical record (EMR) systems may assist clinicians in identifying high-risk hospitalized patients with diabetes who would benefit from intensified cardiorenal protective strategies. Moreover, the incremental improvement of the TyG-NLR model (ΔNagelkerke R² = 0.02, LR χ² = 50.8, P < 10^-10^) suggests that this index offers additional prognostic information beyond traditional factors such as hypertension, albumin, and uric acid. These findings support integrating TyG-NLR into comprehensive risk prediction frameworks.

Several limitations should be acknowledged. First, the retrospective single-center design precludes causal inference and may introduce residual confounding, although we adjusted for key demographic and metabolic covariates. Therefore, we avoided drawing strong causal conclusions and interpreted the findings primarily as epidemiological associations. Second, Second, we were unable to include several important factors, such as glycated hemoglobin (HbA1c), physical activity, dietary habits, and medication adherence, which may influence both inflammation–metabolic indices and cardio-renal outcomes. The absence of these variables should be regarded as a potential source of unmeasured confounding, and future studies incorporating these parameters are warranted to verify the robustness of our findings. Although estimated glomerular filtration rate (eGFR) was collected during data acquisition and used to verify the accuracy of chronic kidney disease (CKD)–related diagnoses in our cohort, it was not entered as an independent covariate in the final statistical models. The main reason is that eGFR constituted part of the disease classification framework rather than an exposure or confounder to be adjusted for, and including it could raise concerns about model overadjustment and collinearity with the outcome definition. Third, the cross-sectional assessment of biomarkers and disease severity restricted longitudinal inference; prospective validation is needed to confirm their temporal predictive value. Future research should explore whether dynamic changes in TyG-NLR or CTI during hospitalization or follow-up could predict the onset of cardiovascular or renal events.

## 5 Conclusion

This study demonstrates that both TyG-NLR and CTI are independently associated with the severity of cardio-renal impairment among patients with type 2 diabetes. Among the two, TyG-NLR exhibited a steeper risk gradient and provided a modest incremental predictive value. Prospective, multicenter longitudinal studies are needed to further validate their predictive utility and potential therapeutic implications across diverse populations.

## Data Availability

The raw data supporting the conclusions of this article will be made available by the authors, without undue reservation.
